# High-dimensional regression analysis links magnetic resonance imaging features and protein expression and signaling pathway alterations in breast invasive carcinoma

**DOI:** 10.18632/oncoscience.397

**Published:** 2018-02-26

**Authors:** Michael Lehrer, Anindya Bhadra, Sathvik Aithala, Visweswaran Ravikumar, Youyun Zheng, Basak Dogan, Emerlinda Bonaccio, Elizabeth S. Burnside, Elizabeth Morris, Elizabeth Sutton, Gary J. Whitman, Jose Net, Kathy Brandt, Marie Ganott, Margarita Zuley, Arvind Rao, TCGA Breast Phenotype Research Group

**Affiliations:** ^1^Department of Bioinformatics and Computational Biology, University of Texas MD Anderson Cancer Center, Houston, TX, USA; ^2^Department of Statistics, Purdue University, West Lafayette, IN, USA; ^3^Department of Biostatistics, Emory University, Atlanta, GA, USA; ^4^Department of Radiology, UT Southwestern, Dallas, TX, USA; ^5^Department of Diagnostic Radiology, Roswell Park Cancer Institute, Buffalo, NY, USA; ^6^Department of Radiology, University of Wisconsin—Madison, Madison, WI, USA; ^7^Department of Radiology, Memorial Sloan Kettering Cancer Center, New York, NY, USA; ^8^Department of Radiology, MD Anderson Cancer Center, Houston, TX, USA; ^9^Department of Radiology, University of Miami Health System, Miami, FL, USA; ^10^Department of Radiology, Mayo Clinic, Rochester, MN, USA; ^11^Department of Radiology, University of Pittsburgh, Pittsburgh, PA, USA; ^12^https://wiki.cancerimagingarchive.net/display/Public/TCGA+Breast+Phenotype+Research+Group

**Keywords:** breast invasive carcinoma, MRI, protein expression, signaling pathway analysis, TCGA

## Abstract

**Background:**

Imaging features derived from MRI scans can be used for not only breast cancer detection and measuring disease extent, but can also determine gene expression and patient outcomes. The relationships between imaging features, gene/protein expression, and response to therapy hold potential to guide personalized medicine. We aim to characterize the relationship between radiologist-annotated tumor phenotypic features (based on MRI) and the underlying biological processes (based on proteomic profiling) in the tumor.

**Methods:**

Multiple-response regression of the image-derived, radiologist-scored features with reverse-phase protein array expression levels generated association coefficients for each combination of image-feature and protein in the RPPA dataset. Significantly-associated proteins for features were analyzed with Ingenuity Pathway Analysis software. Hierarchical clustering of the results of the pathway analysis determined which features were most strongly correlated with pathway activity and cellular functions.

**Results:**

Each of the twenty-nine imaging features was found to have a set of significantly correlated molecules, associated biological functions, and pathways.

**Conclusions:**

We interrogated the pathway alterations represented by the protein expression associated with each imaging feature. Our study demonstrates the relationships between biological processes (via proteomic measurements) and MRI features within breast tumors.

## INTRODUCTION

Breast cancer is the most common cancer in women [1], with incidence rates rising since the 1990s [2]. Molecular expression profiling of tumors has been effective in allowing for individualized therapy plans in certain types of breast cancer [3]. Expression of three receptors—estrogen receptor (ER), progesterone receptor (PR), and human epidermal growth factor receptor (HER2)—are routinely used to determine optimal treatment plans for breast cancer patients [4]. PR and ER expression are associated with luminal A and B subtypes of breast cancer, with a lower proliferation index and pathological grade [5]. Disease-free and overall survival is lower in HER2 over-expression and triple negative breast cancers when compared to luminal A and B subtypes [5].

Despite obtaining multiple specimens from percutaneous biopsies as well as analysis of surgical specimens, the temporal and spatial heterogeneity of tumor gene and protein expression cannot be adequately determined [6, 7, 8]. Readily available imaging databases such as The Cancer Imaging Archive (TCIA) are leveraged in order to address the problem of tumor heterogeneity and to predict gene expression and patient responses to therapy based on imaging data [9]. MRI, as well as other modalities, is now used by researchers for extraction of features which correlate with patient responses and gene expression [10-12]. Breast cancer radiomic signatures can potentially predict recurrence when compared with multi-gene assays [13]. Deciphering the associations between imaging features, breast tumor gene/protein expression levels, and patient outcomes holds the potential to guide personalized medicine [12, 14].

High-dimensional variable selection (Supplementary Table S1) is commonly used to analyze relationships between multiple modalities (copy number, expression, etc.) in genomic data. To avoid generating spurious correlations, a number of Bayesian and frequentist approaches have been devised. Bayesian approaches use a sparsity-inducing prior, such as spike-and-slab [15, 16], double-exponential [17], horseshoe [18], horseshoe+[19], or generalized double Pareto prior [20]. Frequentist approaches use penalized regression models: *l*_1_ [18], horseshoe+ [19], generalized double Pareto prior [20], *L*_1_-norm penalty of the LASSO [21], combined l_1_/l_2_ penalty of elastic net [21], or combined *L*_1_ and L_2_-norm penalty of elastic net [22]. These regression models allow us to ignore the loss of statistical efficiency that occurs through correlation structures because they treat all variables as independent [23]. Several approaches to high-dimensional variable selection in highly-correlated datasets have been taken [24-26]. In this study, we used a Bayesian approach to model the correlation structure as previously described [27].

Analyzing a cohort of 82 breast cancer patients included in the TCGA database, we built a model correlating MRI-derived imaging features with proteomics data using a high-dimensional regression approach. Though a previous study of 353 breast cancer patients assessed correlations between 21 imaging traits and mRNA transcript levels [28], to our knowledge our approach has not yet been applied to proteomics data for breast cancer.

## RESULTS

Molecules were found to be significantly correlated to each imaging feature (Supplementary Table S2) with the exception of clumped non-mass internal enhancement. These molecules were obtained through high dimensional regression of the RPPA protein expression data on the imaging features set. For example, the axillary lymphadenopathy feature was found to be directly correlated with expression of EIF4EBP1 and PRDX1, and inversely correlated with RAB25, SHC1, XRCC1, and PARK7. Cell surface receptors associated with imaging features are EGFR, KDR, and PDK1.

IPA analysis was implemented to determine the functional implications of the molecules. The IPA software generated p-values and Z-scores for the IPA Canonical Pathways of each feature (Figure 2), as well as scores for the IPA Diseases and Biological Functions of each feature (Figure 3). The Canonical Signaling Pathways most strongly associated with each imaging feature are summarized in Table 2. The results show that the same proteins are found to be correlated with a specific feature, irrespective of whether the data sets were separated into global or primary features.

**Figure 1:**
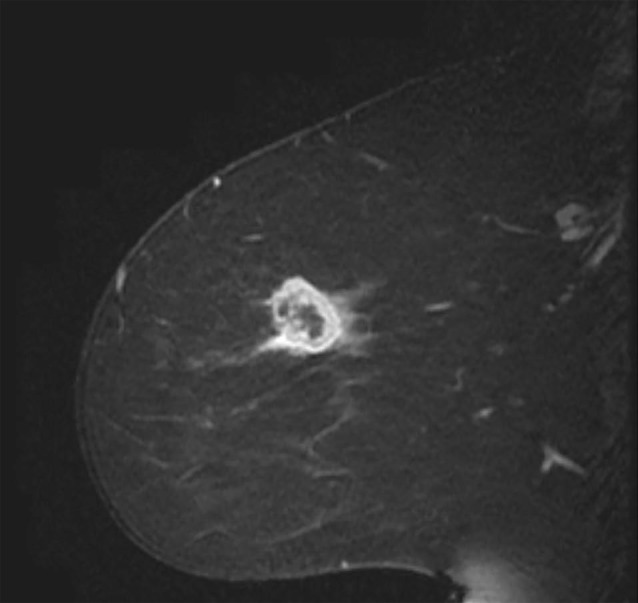
Sagittal T1 post-contrast MRI of a 48-year-old female patient diagnosed with infiltrating ductal carcinoma (ER-, PR-, HER2-) shows an oval rim enhancing mass MRI sequences were obtained from The Cancer Imaging Archive [37].

**Figure 2:**
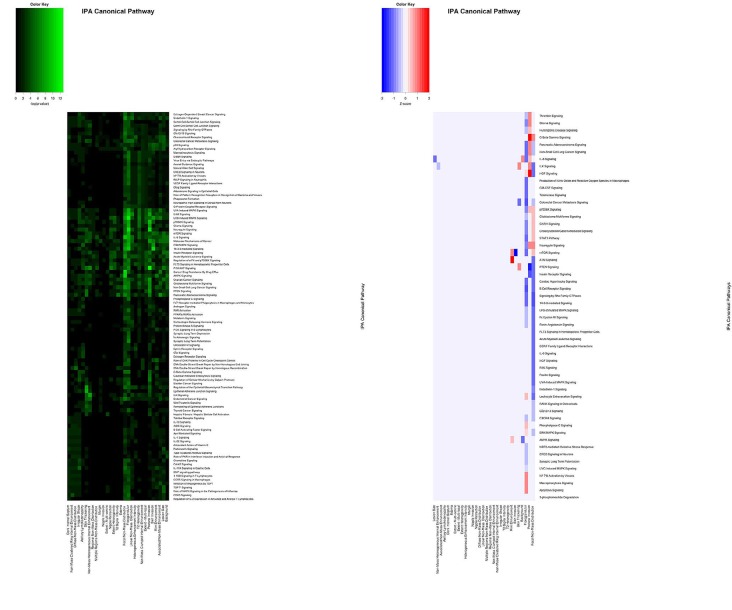
Representative pattern of associations between BRCA imaging features and IPA Canonical Pathways based on (A) p-values and (B) activation Z-scores A subset of the p-values and Z-scores are shown. Values shown are -log(p-value).

**Figure 3:**
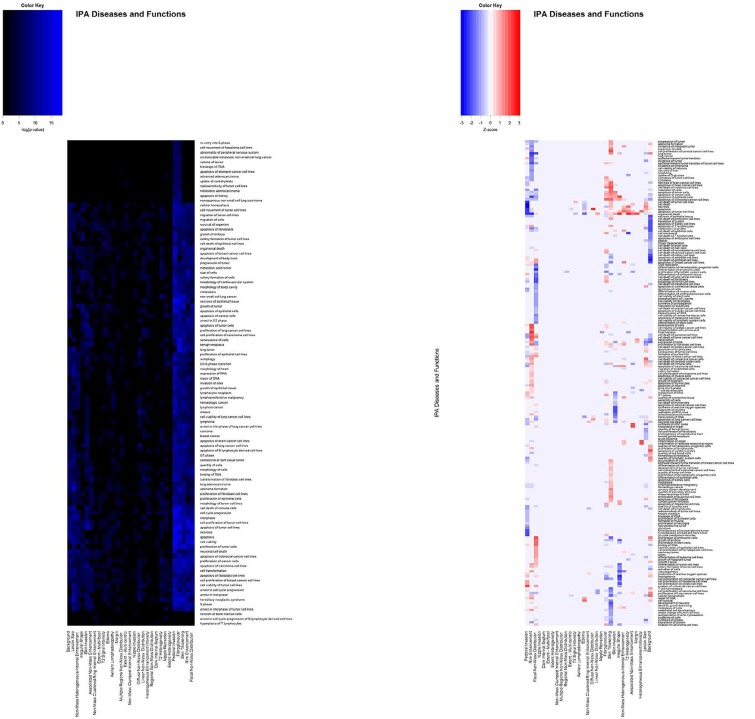
Representative pattern of associations between BRCA imaging features and IPA Diseases and Bio-Functions based on (A) p-values and (B) activation Z-scores A subset of the p-values and Z-scores are shown. Values shown are -log(p-value).

**Table 1:** Patient demographic information Demographics are given for the 82 patients included in this study.

Statistic	
Mean Age at Diagnosis (Range)	53.2(29 - 82)
Median Overall Survival (Months)	41.72
Median Disease-Free Survival (Months)	42.015
Estrogen Receptor (ER) Status (Positive / Negative)	67/ 15
Progesterone Receptor (PR) Status (Positive / Negative)	59/ 23
Infiltrating Lobular Carcinoma	9
Infiltrating Ductal Carcinoma	69
Medullary Carcinoma	1
Other	3

**Table 2:** Radiological features are associated with unique pathway alterations in breast invasive carcinoma Lists of molecules (proteins and post-translational modifications) were analyzed in IPA. Top pathways for each feature are shown with the associated –log (p-value) computed by IPA demonstrating the strength of the association of each imaging feature to each pathway.

3 greatest P-values per imaging feature	1	2	3
*T2 Signal Intensity*	Pancreatic Adenocarcinoma Signaling 6.177	Melanoma Signaling 4.405	Non-Small Cell Lung Cancer Signaling 4.111
*T2 Heterogeneity*	UVB-Induced MAPK Signaling 6.245	EGF Signaling 6.206	ErbB Signaling 5.725
*Skin Thickening*	Epithelial Adherens Junction Signaling 8.957	Regulation of the Epithelial-Mesenchymal Transition Pathway 8.282	Pancreatic Adenocarcinoma Signaling 5.692
*Skin Invasion*	14-3-3-mediated Signaling 10.378	Cell Cycle: G2/M DNA Damage Checkpoint Regulation 7.914	UVB-Induced MAPK Signaling 7.385
*Irregular Shape*	UVC-Induced MAPK Signaling 6.845	EGF Signaling 6.206	STAT3 Pathway 6.112
*Rim Enhancement*	ATM Signaling 8.26	AMPK Signaling 6.385	Cell Cycle: G2/M DNA Damage Checkpoint Regulation 5.037
*Pectoral Invasion*	PI3K/AKT Signaling 12.834	Neuregulin Signaling 10.295	p70S6K Signaling 9.069
*Non-Mass Heterogeneous Internal Enhancement*	ILK Signaling 7.812	PI3K/AKT Signaling 6.646	Endometrial Cancer Signaling 5.511
*Non-Mass Clustered Ring Internal Enhancement*	ATM Signaling 4.078	CDK5 Signaling 3.892	B Cell Receptor Signaling 3.349
*Non-Mass Clumped Internal Enhancement*	DNA Double-Strand Break Repair by Homologous Recombination 3.181	DNA Double-Strand Break Repair by Non-Homologous End Joining 3.181	DNA damage-induced 14-3-3σ Signaling 3.049
*Regional Non-Mass Distribution*	Acute Myeloid Leukemia Signaling 3.745	Cancer Drug Resistance By Drug Efflux 1.94	autophagy 1.853
*Multiple Regions Non-Mass Distribution*	PI3K/AKT Signaling 4.471	IL-8 Signaling 4.068	CD27 Signaling in Lymphocytes 2.311
*Linear Non-Mass Distribution*	ErbB2-ErbB3 Signaling 6.883	ErbB Signaling 6.421	Relaxin Signaling 5.845
*Focal Non-Mass Distribution*	UVC-Induced MAPK Signaling 11.037	Cancer Drug Resistance By Drug Efflux 10.613	AMPK Signaling 10.337
*Diffuse Non-Mass Distribution*	DNA Double-Strand Break Repair by Homologous Recombination 5.395	Role of BRCA1 in DNA Damage Response 3.879	ATM Signaling 3.857
*Nipple Retraction*	PI3K/AKT Signaling 6.112	UVB-Induced MAPK Signaling 4.246	FLT3 Signaling in Hematopoietic Progenitor Cells 4.025
*Nipple Invasion*	Estrogen-mediated S-phase Entry 2.346	Induction of Apoptosis by HIV1 1.949	Lymphotoxin β Receptor Signaling 1.901
*Margin*	Molecular Mechanisms of Cancer 3.746	DNA damage-induced 14-3-3σ Signaling 2.205	GADD45 Signaling 2.205
*Lesion Size*	Hereditary Breast Cancer Signaling 7.625	PI3K/AKT Signaling 5.976	Insulin Receptor Signaling 5.753
*Heterogeneous Enhancement Intensity*	Prolactin Signaling 6.64	Th1 Pathway 6.001	Th1 and Th2 Activation Pathway 5.588
*Fibroglandular*	UVC-Induced MAPK Signaling 13.629	UVB-Induced MAPK Signaling 12.176	Neuregulin Signaling 11.271
*Extent Heterogeneity*	Prostate Cancer Signaling 7.076	UVB-Induced MAPK Signaling 4.546	FLT3 Signaling in Hematopoietic Progenitor Cells 4.325
*Extent - Multi-focal*	CNTF Signaling 4.587	UVB-Induced MAPK Signaling 4.546	EGF Signaling 4.52
*Extent - Multi-centric*	Hepatic Fibrosis / Hepatic Stellate Cell Activation 3.359	Tumoricidal Function of Hepatic Natural Killer Cells 2.346	Coagulation System 2.182
*Edema*	Hereditary Breast Cancer Signaling 4.797	AMPK Signaling 4.425	Endometrial Cancer Signaling 3.607
*Dark Internal Septum*	Huntington's Disease Signaling 3.418	Glucocorticoid Receptor Signaling 3.267	Parkinson's Signaling 2.646
*Background*	Insulin Receptor Signaling 5.753	Molecular Mechanisms of Cancer 5.539	NF-κB Signaling 5.331
*Axillary Lymphadenopathy*	ERK/MAPK Signaling 4.8	EGF Signaling 3.824	Erythropoietin Signaling 3.693
*Associated Non-Mass Enhancement*	Pancreatic Adenocarcinoma Signaling 7.206	UVC-Induced MAPK Signaling 6.399	Cancer Drug Resistance By Drug Efflux 6.194

In order to determine which features were most strongly associated with functional alterations to signaling pathways, agglomerative unsupervised hierarchical clustering was performed on the p-values and Z-scores (Figures 2 and 3). This analysis separated the features into groups based on the strength of their correlations with altered pathway activity and disease functions. The most strongly deregulated IPA Diseases and Biological functions featured activation Z-scores between -3.5 and +3.5 (Supplementary Table S3).

## DISCUSSION

The strength of the associations between imaging features and protein expression, signaling pathways, and biological functions was computed using a sequential analysis of the protein expression data found through RPPA analysis of MRI scans of the TCGA patients. Correlation coefficients for each possible combination of imaging feature and protein were computed using a high-dimensional regression with a Bayesian selection of covariates. Corrected p-values were computed for each correlation coefficient in order to minimize the false discovery rate (FDR). Only the strongest ten percent of significantly-correlated molecules were analyzed using the standardized Core Analysis workflow in IPA, using correlation coefficients in lieu of gene expression values. The IPA analysis provides associations with pathway activity and pathobiology, allowing for hypotheses regarding the relationship between pathway activity at the cellular level and the manifestations of the alterations at the macroscopic, imaging levels. The activation Z-scores computed from the correlation coefficients indicate whether each pathway (or function) is up- or down-regulated by upstream transcription factor activity. A similar approach integrated breast cancer transcriptomics data with imaging features and extended the interpretation with gene set enrichment analysis to identify metagene signatures such as wound response and hypoxia [28]. Our study extends this approach by leveraging the IPA Knowledge Base to interpret the patterns of protein expression associated with each imaging feature.

In our study, we used a stringent two-step method to select the correlations least likely to result from chance association, overcoming a common issue with high dimensional regression analysis. Despite this, the approach we have described is essentially a hypothesis-generation pipeline, and should be interpreted carefully, following in-vivo perturbation experiments in appropriate model systems.

We found that enhancing rim fraction score, a quantitative MRI feature, was shown to be significantly associated with the expression of the long, non-coding RNA HOTAIR [29]. This expression is known to be associated with breast cancer progression and metastasis [30]. The results of the high dimensional regression method used hints at the molecular underpinnings of macroscopic imaging phenotypes. It is known that MRI features correlate with pathologic stage and lymph node involvement [31]. The results found in this study point to multiple significant associations between molecular expression patterns in the tumor cells and how these manifest as MRI phenotypes [32].

## METHODS

### TCGA patient datasets

Eighty-two patients from multiple institutions with de-identified MRIs and reverse-phase protein array (RPPA) expression data were included in this study. All subject data was de-identified prior to the study through inclusion in The Cancer Genome Atlas (TCGA), and was thus exempt from requiring institutional review board approval, following the terms of the TCGA data use agreement. Imaging data was obtained through The Cancer Imaging Archive (TCIA) database. RPPA protein expression data was obtained from the TCGA through Firehose (https://gdac.broadinstitute.org/).

Scores of twenty-nine MRI semantic features were defined by the TCGA Breast Phenotype Research Group [33]. We used the imaging features as defined by the TCGA group to include mass- and non-mass associated features as shown in Table 3. These feature groups include background features, tumor related features, tumor dimensional features, features associated with the morphology of the non-mass enhancing lesion, and T2-weighted MR acquisition features.

**Table 3:** List of imaging features

Feature Group	Features
*Background*	Background Enhancement Fibroglandular
*Tumor Features*	Irregular Shape Heterogeneous Enhancement Intensity Dark Internal Septum Rim Enhancement Margin
*Tumor Dimensions*	Lesion Size Multicentric Extent Multifocal Extent Heterogeneity Extent
*Associated Features*	Pectoral Invasion Nipple Invasion Skin Invasion Axillary Lymphadenopathy Edema Skin Thickening Nipple Retraction
*Morphology of Non-Mass Enhancing Lesions*	Associated Non-Mass Enhancement Non-mass Clumped Internal Enhancement Non-mass Clustered Ring Internal Enhancement Non-mass Heterogeneous Internal Enhancement Diffuse Non-Mass Distribution Focal Non-Mass Distribution Linear Non-Mass Distribution Multiple Regions Non-Mass Distribution Regional Non-Mass Distribution
*Associated with T2 Weighted MR Acquisition*	Heterogeneity Signal Intensity

In order to ensure that the effects of each individual feature were appropriately described, the feature set was split into three subsets: one set with only the 8 mass-associated features, one with only the 21 global features, and an aggregate set with all 29 features. The features were isolated in order to determine if there were any significant proteins, associated pathways, or biological functions that appeared in purely global or mass-associated-only feature sets.

### Statistical analysis

#### High dimensional regression

High dimensional regression was done in Matlab using the joint Bayesian selection of covariates developed by Bhadra and Mallick [27]. In this analysis, the independent variables were the imaging features, and the molecules (proteins and phospho-proteins) were the response variables. This arrangement allowed the expression of each protein to be correlated with the expression of many other proteins.

#### Multiple-testing correction

Multiple testing correction was employed to control the false-discovery rate (FDR) by sequentially designating p-value thresholds [34]. First, the posterior probabilities of the covariates were thresholded at an FDR of 0.25, giving a sparse set of predictors (imaging variables). Second, t-tests were performed using “no-association” as the null hypothesis and “non-zero association” as the alternative hypothesis. The t-tests were computed between each combination of imaging features and molecules in the RPPA dataset. Correlation coefficients with p-values in the 10th percentile and that were less than 0.05 after adjustment for multiple comparisons were considered statistically significant. This approach is similar to that used to discern the relative impact of copy number alterations on messenger RNAs and microRNAs in glioblastoma [30].

#### Pathway analysis

Pathway analysis was performed on each of the three data sets (based on the image feature subsets) using the “Core Analysis” feature in the IPA software [35]. For the purposes of this analysis, regression correlation coefficients served as expression values. P-values and activation Z-scores were computed internally in IPA as previously described.

#### Hierarchical clustering

Agglomerative unsupervised hierarchical clustering of p-values and activation Z-scores was carried out the using the “Stats” package in R. Euclidean distance matrices were computed and Ward's method was minimized within-cluster variance [36].

## SUPPLEMENTARY MATERIALS TABLES


