# Supplementary material for: High-dimensional regression analysis links magnetic resonance imaging features and protein expression and signaling pathway alterations in breast invasive carcinoma
Source: Oncoscience. 2018 Feb 26;5(1-2):39–48. doi: 10.18632/oncoscience.397 (PMC5854291; doi:10.18632/oncoscience.397)
Supplement: Supplementary file 1 [file oncoscience-05-0039-s001.pdf]

SUPPLEMENTARY MATERIALS

Supplementary Tables 1-3: Please see pages below.

Supplementary Table S1. Description of variables and parameters comprising the high-dimensional regression model.

Symbol	Dimension	Description	Symbol	Dimension	Description
n	scalar	sample size	$\mathbf{G}$	$q \times q$	conditional independence graph
p	scalar	number of predictor variables	$\mathbf{B}_{\boldsymbol{\gamma}}$	$p_{\boldsymbol{\gamma}} \times q$	matrix of regression coefficients
q	scalar	number of response variables	$\mathbf{Y}$	$n \times q$	matrix of responses
p_γ	scalar	number of selected predictor variables	$\mathbf{E}$	$n \times q$	matrix of regression errors
$\boldsymbol{\gamma}$	p	vector of indicators for selecting predictors	$\boldsymbol{\Sigma}_{\mathbf{G}}$	$q \times q$	column covariance for errors
$\mathbf{X}$	$n \times p$	matrix of available predictors	$\mathbf{W}$	$q \times q$	symmetric matrix of edge weights
$\mathbf{X}_{\boldsymbol{\gamma}}$	$n \times p_\gamma$	matrix of selected predictors	$\mathbf{T}_{\boldsymbol{\gamma}}$	$n \times q$	matrix of marginal data distribution

Supplementary Table S2. Significantly-associated RPPA molecules. Multiple response regression was applied to the combined BRCA imaging feature set and RPPA dataset from 82 patients, and the results were filtered to include only molecules significantly correlated with each imaging feature after multiple-testing correction.

Imaging Feature	Positively correlated proteins	Negatively correlated proteins
T2 Signal Intensity	RB1, YAP1, BAD, PDCD4, KDR	RAD51, BAK1, ERBB3
T2 Heterogeneity	None	ACACA ACACB, FASN, NF2, PIK3CA, PRKCA , MTOR, SQSTM1
Skin Thickening	EIF4EBP1, CASP3, DVL3, CDH1, MYH9, PEA15, RPS6, YBX1, CTNNB1	EGFR, FOXO3, STK11, NOTCH1, RAD51, STMN1, MET, CDKN1B, CHEK1
Skin Invasion	YWHAZ, EIF4EBP1, PRDX1	EIF4EBP1, BAD, CHEK1, CHEK2, PRKCD, AKT1S1, SCD, JUN, MET, RPS6KA1
Irregular Shape	None	ANXA1, EGFR, NDRG1, SRC, YAP1, MAPK14, PEA15
Rim Enhancement	EIF4EBP1, BECN1, CHEK1, CHEK2, EGFR, ERCC1, FOXO3, STK11, MRE11A, SERPINE1, PDCD4, AKT1S1, RICTOR, ABL1, JUN, PRKCA, STMN1, CDKN1A	ACACA, ANXA7, NRG1, ACACA ACACB, MAPK14, BAP1, ESR1, FASN
Pectoral Invasion	YWHAB, YWHAE, MS4A1, ITGA2, CASP8, CAV1, CHEK1, COL6A1, CCND1, EGFR, ERCC1, FOXO3, ERBB3, MYH11, PARP1, PRKCA , RAD51, RB1, SHC1, SNAI1, STMN1, XBP1, MET, STMN1	BRD4, BAP1, DVL3, JAK2, PDK1, RBM15, EIF4G1, MTOR, RPS6KB1, DPP4
Non-Mass Heterogeneous Internal Enhancement	YWHAB, ACVRL1, DIRAS3, FOXO3, FN1, RAB11A RAB11B	TP53BP1, CDH1, GSK3A GSK3B, RPTOR, CTNNB1, MTOR, BRAF, BAP1
Non-Mass Clustered Ring Internal Enhancement	PDK1, TGM2, MAPK14	ABL1
Non-Mass Clumped Internal Enhancement	ATM, YAP1	ERRFI1
Regional Non-Mass Distribution	PDK1, KIT	AKT1 AKT2 AKT3, ADAR, INPP4B, MTOR
Multiple Regions Non-Mass Distribution	PIK3R1 PIK3R2	EIF4EBP1, BCL2L1, PXN
Linear Non-Mass Distribution	BRAF, CCNE1	AKT1 AKT2 AKT3, ERBB3, PTPN11, JUN
Focal Non-Mass Distribution	EIF4EBP1, RAF1, CHEK1, EGFR, FOXO3, GSK3A GSK3B, NDRG1, PIK3R1 PIK3R2, AKT1S1, RICTOR, SRC, STMN1, YBX1	TP53BP1, ARAF, ACACA, ADAR, ATM, BRD4, COG3, MAPK1, FASN, GATA3, INPP4B, MAPK9, XRCC5, PDK1, RAB25, AKT1 AKT2 AKT3
Diffuse Non-Mass Distribution	MAPK14	BRCA2, MRE11A, MET, CDKN1B
Nipple Retraction	MYH11, PIK3CA , RICTOR	EIF4EBP1, DPP4, MTOR, CDKN1B
Nipple Invasion	ESR1, DIABLO, XRCC1	GUSP4, PDCD4, RPS6
Margin	PRKAA1, INPP4B, PEA15	ASNS, CASP7, CCNE1, CDH1, FOXM1, IGFR1
Lesion Size	CHEK2, CCNB1, GUSP4, FOXM1, MSH2, MSH6, NOTCH1, EIF4EBP1, TSC1, EIF4G1,	BCL2, PARK7, ESR1, MAPK8, NRAS, COL6A1,

	ADAR, PRKAA1, GSK3A GSK3B, YBX1, EEF2	DIRAS3, RB1, SRC, JUN
Heterogeneous Enhancement Intensity	IRF1, ERCC1	PGR, PTPN11, STAT3, MAPK14
Fibroglandular	PECAM1, ITGA2, CHEK2, STK11, MRE11A, NRAS, PARP1, PRKCD, SCD, SETD2, SRSF1, STMN1, MET, TP53	EIF4EBP1, BCL2A1, EGFR, ERCC5, MAPK9, MAP2K1, NDRG1, PRKCB, RPS6, RPS6KA1, MAPK1 MAPK3, PIK3R1 PIK3R2, RPS6KA1, TP53BP1
Extent Heterogeneity	CCNE2, FOXM1	BAD, MTOR, GSK3A GSK3B, PTPN11
Extent - Multi-focal	PIK3CA, RPS6KB1	TGM2, SRC
Extent - Multi-centric	CLDN7	BAX, SERPINE1, PDK1
Edema	CCND1, FOXO3, CDH2, PEA15, RAD51, TIGAR, SQSTM1	ARID1A
Dark Internal Septum	HSPA1A, MAPK8, KIT	None
Background	EIF4EBP1, BRAF, GAPDH, GSK3A GSK3B, TSC1, KDR, SQSTM1, TUBA1B, CHEK2, MSH2	BID, MS4A1, CASP8, ERCC5, FOXO3, ERBB3, IRS1, RB1, RAB11A RAB11B, COL6A1, ERCC5, HSPA1A
Axillary Lymphadenopathy	EIF4EBP1, PRDX1, SRC	RAB25, SHC1, XRCC1, PARK7
Associated Non-Mass Enhancement	PRKAA1, MAPK9, BCL2, MAPK9, EEF2K	GSK3A GSK3B, ARAF, CCNE1, FOXM1, PIK3CA, SCD, TP53, SCD, TSC1, TGM2, CDK1, CHEK1, CHEK2

Supplementary Table S3. Imaging features are associated with unique biological functions in breast invasive carcinoma. Proteins and phospho-proteins with expression significantly correlated with imaging features were analyzed using Ingenuity Pathway Analysis. Values shown are –log (Z-score).

Imaging Feature	Positively-correlated Diseases and Bio-Functions	Negatively-correlated Diseases and Bio-Functions
T2 Signal Intensity	cell death of tumor cell lines 0.881<br>cell proliferation of tumor cell lines 0.462<br>cell viability 0.38	organismal death -0.92<br>apoptosis -0.184<br>abnormal bone density 0
T2 Heterogeneity	organismal death 2.39<br>necrosis 1.69<br>apoptosis of tumor cell lines 1.309	cell viability of tumor cell lines -2.207<br>cell movement -2.12<br>cell cycle progression -1.896
Skin Thickening	cell movement of fibroblasts 2.228<br>quantity of tumor 2.219<br>cell proliferation of cervical cancer cell lines 1.98	development of neurons -1.949<br>cell viability of lung cancer cell lines -1.94<br>development of body trunk -1.815
Skin Invasion	apoptosis of tumor cells 1.446<br>organismal death 1.163<br>apoptosis of cancer cells 1.154	interphase -2.321<br>S phase -1.981<br>outgrowth of neurites -1.969
Irregular Shape	organismal death 2.38<br>apoptosis of epithelial cells 1.964<br>apoptosis of colorectal cancer cell lines 1.131	angiogenesis -2.225<br>cell proliferation of tumor cell lines -2.222<br>cell proliferation of breast cell lines -1.987
Rim Enhancement	Viral Infection 2.435<br>cell viability 2.4<br>cell viability of tumor cell lines 2.255	lung tumor -2.296<br>organismal death -2.275<br>apoptosis of epithelial cells -1.964
Pectoral Invasion	neoplasia of tumor cell lines 3.116<br>migration of tumor cell lines 2.761<br>cell movement of tumor cell lines 2.733	necrosis -3.09<br>cell death -2.855<br>organismal death -2.443
Non-Mass Heterogeneous Internal Enhancement	autophagy of cells 1.982<br>autophagy 1.961<br>apoptosis of tumor cell lines 1.553	cell proliferation of kidney cell lines -1.982<br>cell cycle progression -1.561<br>vasculogenesis -1.436
Non-Mass Clustered Ring Internal Enhancement	cell movement of tumor cell lines 1.93<br>migration of cells 1.731<br>neuronal cell death 0.886	transmembrane potential of mitochondria -1.974 survival of organism -1.946<br>cell death of lymphocytes -0.762

Non-Mass Clumped Internal Enhancement	None	None
Regional Non-Mass Distribution	cell viability of lymphatic system cells 0.293	proliferation of lymphatic system cells -0.686
Multiple Regions Non-Mass Distribution	None	None
Linear Non-Mass Distribution	organismal death 0.933<br>necrosis of epithelial tissue 0.205<br>expression of RNA 0.029	quantity of cells -1.899<br>mitosis -1.067<br>transformation of fibroblast cell lines -0.972
Focal Non-Mass Distribution	cell viability of lung cancer cell lines 2.19<br>proliferation of embryonic cells 2<br>lymphoid cancer 1.977	differentiation of embryonic cells -2.171<br>autophagy of tumor cell lines -1.982<br>cell death of fibroblasts -1.932
Diffuse Non-Mass Distribution	apoptosis 2.17<br>apoptosis of lung cancer cell lines 1.109<br>migration of cells 1.041	cell viability of tumor cell lines -2.189<br>quantity of cells -0.75
Nipple Retraction	migration of cells 1.608<br>quantity of protein in blood 1.131<br>growth of tumor 0.927	Viral Infection -1.021<br>metabolism of protein -0.263
Nipple Invasion	apoptosis 0.252	None
Margin	organismal death 1.995<br>transcription 1.118<br>expression of RNA 0.837	cell cycle progression -1.943<br>quantity of cells -1.896<br>cell viability -1.849
Lesion Size	cell death of connective tissue cells 1.747<br>cell cycle progression 1.492<br>interphase 1.492	microtubule dynamics -2.519<br>organization of cytoplasm -2.519<br>organization of cytoskeleton -2.519
Heterogeneous Enhancement Intensity	inflammation of organ 1.964<br>inflammation of absolute anatomical region 1.955<br>apoptosis of tumor cell lines 1.811	cell proliferation of tumor cell lines -1.715<br>cell cycle progression -1.387<br>T cell development -1.039
Fibroglandular	apoptosis of sarcoma cell lines 2.214<br>apoptosis of lymphoma cell lines 2.184<br>autophagy 2.065	proliferation of B lymphocytes -2.426<br>cell movement of fibroblasts -2.375<br>tumorigenesis of benign tumor -2.19
Extent Heterogeneity	None	None
Extent - Multi-focal	None	None
Extent - Multi-centric	None	migration of cells -0.592<br>apoptosis -0.547<br>cellular homeostasis -0.343
Edema	cell movement of tumor cell lines 1.959	organismal death -1.795

	invasion of cells 1.948<br><br>cell proliferation of tumor cell lines 1.808	transcription of RNA -1.164<br><br>cell death -0.839
Dark Internal Septum	None	None
Background	apoptosis of cardiomyocytes 1.949<br><br>cell viability of tumor cell lines 1.758<br><br>cellular homeostasis 1.658	cell death of epithelial cell lines -2.57<br><br>apoptosis of epithelial cell lines -2.388<br><br>cell death of kidney cell lines -2.156
Axillary Lymphadenopathy	survival of organism 0.152<br><br>organismal death 0.046<br><br>necrosis 0.023	quantity of cells -1.414<br><br>migration of cells -0.747<br><br>cell transformation -0.587
Associated Non-Mass Enhancement	hypoplasia of organ 1.982<br><br>synthesis of nitric oxide 1.98<br><br>organismal death 1.643	cell cycle progression -1.85<br><br>synthesis of DNA -1.623<br><br>S phase -1.589
